# Filament Negative Regulator *CDC4* Suppresses Glycogen Phosphorylase Encoded *GPH1* That Impacts the Cell Wall-Associated Features in *Candida albicans*

**DOI:** 10.3390/jof8030233

**Published:** 2022-02-26

**Authors:** Wei-Chung Lai, Hsiao-Chi Hsu, Chun-Wen Cheng, Shao-Hung Wang, Wan Chen Li, Po-Szu Hsieh, Tzu-Ling Tseng, Ting-Hui Lin, Jia-Ching Shieh

**Affiliations:** 1 Department of Biomedical Sciences, Chung Shan Medical University, Taichung City 40201, Taiwan; workman_pum250@hotmail.com (W.-C.L.); cookiechi413@gmail.com (H.-C.H.); wanwan9121@hotmail.com (W.C.L.); catstyle0626@hotmail.com (P.-S.H.); linda740410@hotmail.com (T.-L.T.); thlin@csmu.edu.tw (T.-H.L.); 2 Institute of Medicine, Chung Shan Medical University, Taichung City 40201, Taiwan; cwcheng@csmu.edu.tw; 3 Department of Microbiology, Immunology and Biopharmaceuticals, National Chiayi University, Chiayi 60004, Taiwan; shwang@mail.ncyu.edu.tw; 4 Department of Medical Research, Chung Shan Medical University Hospital, Taichung City 40201, Taiwan; 5 Immunology Research Center, Chung Shan Medical University, Taichung City 40201, Taiwan

**Keywords:** *Candida albicans*, *CaCDC4*, *GPH1*, morphogenesis, cell wall

## Abstract

We have previously identified *Candida albicans GPH1* (orf19.7021) whose protein product was associated with *C. albicans* Cdc4. The *GPH1* gene is a putative glycogen phosphorylase because its *Saccharomyces cerevisiae* homolog participates in glycogen catabolism, which involves the synthesis of β-glucan of the fungal cell wall. We made a strain whose *CaCDC4* expression is repressed, and *GPH1* is constitutively expressed. We established a *GPH1* null mutant strain and used it to conduct the in vitro virulence assays that detect cell wall function. The in vitro virulence assay is centered on biofilm formation in which analytic procedures are implemented to evaluate cell surface hydrophobicity; competence, either in stress resistance, germ tube formation, or fibronection association; and the XTT-based adhesion and biofilm formation. We showed that the constitutively expressed *GPH1* partially suppresses filamentation when the *CaCDC4* expression is repressed. The *C. albicans* Gph1 protein is reduced in the presence of *Ca*Cdc4 in comparison with the absence of *Ca*Cdc4. Compared with the wild-type strain, the *gph1*Δ/*gph1*Δ mutant displayed a reduction in the capability to form germ tubes and the cell surface hydrophobicity but an increase in binding with fibronectin. Compared with the wild-type strain, the *gph1*Δ/*gph1*Δ mutant showed a rise in adhesion, the initial stage of biofilm formation, but displayed a similar capacity to form a mature biofilm. There was no major impact on the *gph1*Δ/*gph1*Δ mutant regarding the conditions of cell wall damaging and TOR pathway-associated nutrient depletion. We conclude that *GPH1*, adversely regulated by the filament suppressor *CDC4*, contributes to cell wall function in *C. albicans*.

## 1. Introduction

The opportunistic human fungal pathogen *Candida albicans* [1] is a member of the normal microflora on mucosal surfaces in healthy persons [2] but can cause vulvovaginal candidiasis in women [3,4] and oral [5,6] and systemic candidiasis in debilitated and immunocompromised patients [7,8,9,10]. *C. albicans* can grow in a wide variety of morphological forms, from the ellipsoid blastospore to various filamentous types [11,12,13,14]. A great effort has been made to reveal the underlying mechanism of *C. albicans* morphogenesis because it is proven to be coupled with virulence and pathogenesis [15,16,17,18]. However, research advancement has been hampered due to *C. albicans* being a natural diploid with a noncanonical sexual cycle [19,20,21,22]. Still, several positive and negative signaling pathways that control morphological transition have been discovered in *C. albicans* [23,24,25]. Additionally, cyclin-dependent kinases and their associated cyclins with their regulators have been found to control morphological plasticity in *C. albicans* [26,27]. Curiously, we and others have recently found that some key cell cycle genes conserved throughout evolution play an essential role in the cell cycle but influence morphogenesis in *C. albicans* [28,29,30,31,32,33], including the couple cell cycle and morphogenesis [34,35,36,37].

We and others have uncovered that *CaCDC4* suppresses filamentation in *C. albicans* [28,33]. The *Ca*Cdc4 has the WD40–repeat and F-box domains, whose homologs participate in binding with Skp1, one of the components of the Skp1-Cdc53/Cul1-F-box (SCF) complex, and the substrate [38], respectively. The *CaCDC4* encodes a conventional F-box protein of SCF ubiquitin ligase [39], designated SCF*^Ca^*^Cdc4^. Notably, we revealed that the domains of F-box and WD40-repeat in the *Ca*Cdc4 are critical for filamentous development and repress flocculation [40]. Other than filamentation [41,42,43], flocculation is closely connected to biofilm formation [44,45,46]. Indeed, we found that *CaCDC4* negatively regulates biofilm formation in *C. albicans* [47]. By affinity purification, we identified several novel *Ca*Cdc4-interactors [48], among which are Gph1 and Thr1. In the budding yeast *Saccharomyces cerevisiae*, while the *THR1* gene encodes a homoserine kinase that participates in the threonine biosynthesis pathway of [49,50]. Intriguingly, we found that *C. albicans THR1* links *GCN4* and *CaCDC4* to control morphogenesis with the stress response and nutrient sensing [51], indicating that the morphogenesis is intertwined with environmental factors. In *S. cerevisiae*, *GPH1* encodes a glycogen phosphorylase essential for the breakdown of glycogen polysaccharide to glucose-1-phosphate and glucose [52], which feed into glycolysis.

Glucose-1-phosphate can be converted into UDP-glucose in the presence of UTP by Upg1 [53]. UDP-glucose can be directed to either the storage carbohydrate glycogen by Glg1 and Glg 2 [54] or glucose by the trehalose synthase complex [54,55]. Importantly, UDP-glucose can be used as a substrate to become the structural carbohydrate β-glucan, one of the major components of the cell wall [56,57]. Hence, *GPH1* appears to be involved in the synthesis of β-glucan and energy conversion in *S. cerevisiae* and may be shared in fungi including *C. albicans.* Several lines of evidence point to the association of *GPH1* function with the cell wall in *C. albicans*. Gph1 was found to be non-covalently linked to the cell wall that is enriched in hyphal cells of *C. albicans* [58]. *C. albicans* cells treated with fluconazole exhibited a 3.5-fold up-regulated expression of *GPH1* [59]. *GPH1* was found to be under the Ndt80-dependent transcriptional control for biofilm formation in *C. albicans* [60]. *GPH1* was found to be a common output of Cph2, which is required for the optimal expression of some hypoxia-responsive genes in glycolysis and the citric acid cycle and the regulatory circuit for gastrointestinal (GI) colonization [61,62,63]. In this report, we found that the level of Gph1 protein was decreased in conditions when the expression of *CaCDC4* was de-repressed, and the filaments caused by the repressed *CaCDC4* expression could be overcame by the constitutive expression of *GPH1* in *C. albicans.* To investigate the role of *GPH1* in *C. albicans*, we generated a *gph1* null mutant. Cells of the *gph1* null mutant were maintained as the yeast form without growth defect, but they were aggregated after prolonged culture. We made a strain where the expression of *GPH1* is under the Tet-on control. In the induced condition, cells of the strain exhibited no morphological changes and peculiarly accumulated glycogen compared with those of the *gph1* null mutant and wild-type. The *gph1* null mutant did not appear to show growth impairment in a variety of cell wall damaging agents, carbon sources, and amino acid depleted conditions. However, the *gph1* null mutant showed a decrease in its cell surface hydrophobicity and its ability to form a germ tube in the hypha-induced condition. Conversely, the *gph1* null mutant exhibited an increase in its ability in either binding with fibronectin or adhesion but made no impact on biofilm formation. Hence, *GPH1* negatively modulated by the filament suppressor *CDC4* is involved in cell wall function in *C. albicans.*

## 2. Materials and Methods

### 2.1. General Manipulation, Media, and Growth Conditions

The *E. coli* strain DH5α was used for regular manipulation of the plasmids. All *C. albicans* strains (Table 1) were derived from either the clinically isolated wild-type strain SC5314 [64] or the auxotrophic strain BWP17 (*arg4/arg4 his1/his1 ura3/ura3*) [65]. The routine usage of media and growth conditions for the strains of *E. coli* and *C. albicans* were performed as described previously [66]. The pH of the SD medium with or without agar was adjusted to 7 by 100 mM HEPES after autoclaving because the default SD medium is acidic, which can suppress the filamentation of *C. albicans*. The *E. coli* strain DH5α was transformed with plasmid DNA by CaCl_2_ as described [67] or by electroporation [68]. *C. albicans* strains were transformed using the LiAc/PEG/ssDNA method [69] or electroporation [70].

### 2.2. Strain Usage and Construction

To enable constitutive expression of *GPH1* in *C. albicans* carrying the expression-repressible *CaCDC4*, the coding sequence of the *GPH1* gene was PCR-amplified from genomic DNA of the *C. albicans* wild-type strain SC5314 [64])with a pair of primers, CaGPH1-XhoI-F and CaGph1-XhoI-R (Table 2) and cloned into the plasmid vector p6HF-*ACT1*p [71] to generate p6HF-*ACT1*p-*GPH1* capable of constitutively expressing *GPH1.* The *CaCDC4* expression repressible strain *CaCDC4 M3*/− [40] (Table 1), whose *CaCDC4* expression is repressed with 2.5 mM methionine and cysteine (Met/Cys) [72], was used to introduce the *Nco*I-linearized plasmid p6HF-*ACT1*p-*GPH1*, along with the empty plasmid p6HF-*ACT1*p and p6HF-*ACT1*p-*CaCDC4* (51) targeting and integrating at the *RPS1* locus to generate *CaCDC4 M3*/−|*GPH1*, *CaCDC4 M3*/−|p6HF-*ACT1*p, and *CaCDC4 M3*/−|*CaCDC4*, respectively (Table 1). Moreover, *GPH1* was deleted in the *C*. *albicans* wild-type strain SC5314 with the *CaSAT1*-flipper method [73]. Briefly, both the upstream and downstream regions of *GPH1* were amplified with primer pairs CaGPH1-U-F_KpnI/CaGPH1-U-R_XhoI and CaGPH1-D-F_SacII/CaGPH1-D-R_SacI, respectively (Table 2), and with template DNA of the genomic DNA extracted from SC5314. These were consecutively cloned into plasmid pSFS2A with a *CaSAT1*-flipper cassette at *Kpn*I/*Xho*I and *Sac*II/*Sac*I sites to make plasmid pSF2A-*gph1*Δ. A cassette freed from pSF2A-*gph1*Δ using *Kpn*I/*Sac*II was introduced into SC5314 and was selected for nourseothricin positive (Nou^+^) *GPH1*/*gph1*ΔSF, following the *CaSAT1*-popped out by induction in YP–maltose (the glucose is replaced with maltose in YPD) to make *gph1* heterozygous null mutant, *GPH1/gph1*Δ. The cassette pSF2A-*gph1*Δ was introduced into *GPH1/gph1*Δ and selected for Nou^+^ (*gph1*ΔSF/*gph1*Δ), following the *CaSAT1*-popped out by induction in YP–maltose for *FLP/FRT* recombination to generate *gph1* homozygous null mutant, *gph1*Δ*/gph1*Δ. To make a *GPH1* reintegrated strain, the DNA cassettes were PCR-amplilied with primer pairs CaGPH1-U-F_KpnI/GPH1-D-XhoI-R and CaGPH1-D-F_SacII/CaGPH1-D-R_SacI, respectively (Table 2), and with template DNA of the SC5314 genomic DNA. These were subsequently cloned into plasmid pSFS2A to become pSF2A-*gph1*, which contains a *CaSAT1*-flipper cassette flanked with the *GPH1* upstream region plus the *GPH1* ORF and *GPH1* downstream region. A cassette freed from pSF2A-*gph1* using *Kpn*I/*Sac*II was introduced into *gph1*Δ*/gph1*Δ and selected for nourseothricin positive (Nou^+^) *gph1*Δ/*gph11*Δ+*GPH1*-*SAT1-FLIP*, following the *CaSAT1*-popped out by induction in YP–maltose to make the *GPH1* complement strain *gph1*Δ*/gph1*Δ+*GPH1*. The strain *gcn4*Δ/*gcn4*Δ was created as previously described [74].

**Table 1:** *Candida albicans* strains used in this study.

Name of the Strain	Parental Strain	Genotype	Source
SC5314		Wild-type strain	[64]
BWP17		*ura3*::imm434*/ura3*::imm434 *iro1/iro1*::imm434 *his1*::*hisG/his1*::*hisG arg4*::*hisG/arg4*::*hisG*	[65]
*CaCDC4 M3*/−	BWP17	*Cacdc4*Δ::*dpl200/Cacdc4*::pFA-*HIS1*-*MET3*p-*CaCDC4*	[40]
*CaCDC4 M3*/−|p6HF-*ACT1*p	*CaCDC4 M3*/−	*Cacdc4*Δ::*dpl200*/*Cacdc4*::pFA-*HIS1*-*MET3*p-*CaCDC4* *RPS1/rps1*::p6HF- *ACT1*p	This study
*CaCDC4 M3*/−|*CaCDC4*	*CaCDC4 M3*/−	*Cacdc4*Δ::*dpl200*/*Cacdc4*::pFA-*HIS1*-*MET3*p-*CaCDC4* *RPS1/rps1*Δ::p6HF-*ACT1*p-*CaCDC4*	This study
*CaCDC4 M3*/−|*GPH1*	*CaCDC4 M3*/−	*Cacdc4*Δ::*dpl200*/*Cacdc4*::pFA-*HIS1*-*MET3*p-*CaCDC4* *RPS1/rps1*Δ::p6HF-*ACT1*p-*GPH1*	This study
*gcn4*Δ/*gcn4*Δ	SC5314	*gcn4*::*FRT/gcn4*::*FRT*	[74]
*GPH1*/*gph1*ΔSF	SC5314	*GPH1/gph1*Δ::*SAT1-FLIP*	This study
*GPH1*/*gph1*Δ	*GPH1*/*gph1*ΔSF	*GPH1/gph1*Δ::*FRT*	This study
*gph1*ΔSF/*gph1*Δ	*GPH1*/*gph1*Δ	*gph1*Δ::*SAT1-FLIP/gph1*Δ::*FRT*	This study
*gph1*Δ/*gph11*Δ	*gph1*ΔSF/*gph1*Δ	*gph1*Δ::*FRT/gph1*Δ::*FRT*	This study
*gph1*Δ/*gph11*Δ+*GPH1*-*SAT1-FLIP*	*gph1*Δ/*gph1*Δ	*gph1*Δ::*FRT/gph1*Δ::*FRT*::*GPH1*-*SAT1-FLIP*	This study
*gph1*Δ/*gph11*Δ+*GPH*	*gph1*Δ/*gph11*Δ+*GPH1*-*SAT1-FLIP*	*gph1*Δ::*FRT/gph1*Δ::*FRT*::*GPH1*	This study
Tet-on-*GPH1*	SC5314	*ADH1/adh1*::*P_TET_-GPH1-SAT1*	This study

**Table 2:** Synthetic oligonucleotide primers used in this study.

Name	Sequence (5′→3′) ^1^
CaGPH1-U-F_KpnI	CGGGGTACCCCACCTAACTAATAACTATTGC
CaGPH1-U-R_XhoI	CCGCTCGAGGGGTAAGATAATCCATTGGC
CaGPH1-D-F_SacII	TCCCCGCGGGAAAGTAAGACAACGAGCGA
CaGPH1-D-R_SacI	CTAGGAGCTCCTTAGCTGAGTTAGGATCTG
GPH1-D-XhoI-R	GGGCTCGAGTCTTTCTCTCCCTTCATTGC
CaGPH1-XhoI-F (p6HF-*ACT1*p)	CCGCTCGAGATGCCAATGGATTATCTTACC
CaGph1-XhoI-R (p6HF-*ACT1*p)	CCGCTCGAGCTAAACATTGGATGGTTCAAC
GPH1-probe-F	CTGATTTAGATCAAGTGGCTGA
GPH1-probe-R	GACGAATGTAATGGCAGAGTT
front of GPH1-F_SpeI	GGACTAGTATGCCAATGGATTATCTTACC
front of GPH1-R_SpeI	GGACTAGTAACCCGTAACCCCAACCAC
CaACT1-F	ACGGTGAAGTTGCTGCTTTA
CaACT1-R	GCATTTCTTGTTCGAAATCC

^1^ Restriction enzyme sites are shaded in grey.

### 2.3. Nucleic Acid Extraction and PCR Analysis

*Candida albicans* cells were grown to the mid-log phase, and genomic DNA was isolated using a MasterPure™ Yeast DNA Purification Kit (Epicentre, Madison, WI, USA), following the manufacturer’s instructions, as described previously [35]. The total RNA derived from cells cultured to the mid-log phase was extracted using a MasterPure^TM^ Yeast RNA Purification Kit (Epicentre, Madison, WI, USA), following the manufacturer’s instructions, as described previously [51]. Subsequently, 5 μg of total RNA was used to generate cDNA using a SuperScript III Reverse Transcriptase Kit (Invitrogen, Carlsbad, CA, USA), following the manufacturer’s instructions. The cDNA was then subjected to PCR with a pair of *GPH1*-specific primers, the front of GPH1-F_SpeI, and the front of GPH1-R_SpeI (Table 2), annealing the downstream of the *GPH1* coding sequence with a predictive product of 623 bp. The primers CaACT1-F and CaACT1-R were used to generate a *C. albicans ACT1*-specific product that was used as a loading control. To confirm the correctness of the *GPH1* deletion strain, Southern blotting analyses with DIG-labelled probe amplified by a pair of primers, GPH1-probe-F and GPH1-probe-R (Table 2), were performed as previously described [74].

### 2.4. Protein Extraction and Western Blotting

The total protein was extracted from the *C. albicans* cells, as described previously (74). The protein was partially purified from cells containing the p6HF-*ACT1*p plasmid with the open reading frame of the gene integrated at *RPS1* capable of generating a tagged (6×His and FLAG) protein using Ni^2+^-NTA-agarose beads (Qiagen, Germantown, MD, USA), as previously described [75]. Precipitated proteins were resolved using 10% SDS-PAGE and transferred electrophoretically to PVDF membranes (Pall Corporation, Port Washington, NY, USA). They were then probed with a polyclonal antibody to FLAG (Sigma) in a 1:2000 dilution and visualized using a SuperSignal West Pico Chemiluminescent Substrate Kit (Pierce). The proteins detected were recorded with a Luminescent Image Analyzer (FUJIFILM LAS-1000) and analyzed by ImageGauge 3.46 and L Process v 1.96 (FUJIFILM). ImageJ (National Institutes of Health, Bethesda, MD, USA) was used to quantify the levels of proteins.

### 2.5. Germ Tube Formation Assay

The morphological plasticity of *C. albicans* plays a vital role in biofilm maturation, as previously discussed. Germ tube formation is a prerequisite of the development of hyphal and pseudohyphal forms, and hence the length of the germ tube under hyphal induction condition is used for the assessment of yeast-to-hypha transition. To promote germ tube formation, 1 × 10^6^ *C. albicans* cells/mL are transferred into the cell culture medium RPMI 1640 supplemented with 10% (*v*/*v*) fetal calf serum (FCS), 2 mM L-glutamine, penicillin (100 U/mL), and streptomycin (100 μg/mL) and seeded into a 24-well plate. After 1 h incubation at 37 °C, cells were visualized and recorded, and the germ tube length was determined with the Photoshop 6 software.

### 2.6. Cell Surface Hydrophobicity Assay

Cell surface hydrophobicity (CSH) was measured using the microbial adhesion assay to hydrocarbons (MATH) [76]. The assay was conducted as previously described [77]. Briefly, *C. albicans* cells grown to the mid-log phase at 30 °C were collected and washed twice with PBS. The cell suspension with an OD_600_ between 0.4 and 0.5 was set up in PBS (A0); 3 mL of the cell suspension was overlaid with 0.4 mL of the hydropho-bichydrocarbon, n-hexadecane (SIGMA, H6703). Following robust vortexing, the phases were left to separate for 10 min at 30 °C, and the OD_600_ of the aqueous phase was quantified (A1). The percentage of hydrophobicity is calculated as follows: hydrophobicity (%) = [1 − (A1/A0)] × 100.

### 2.7. Fibronectin (FN)-C. albicans Association Assay

To specifically assess the binding of fibronectin with *C. albicans* cells, *C. albicans* cells (1 × 10^6^) from the mid-log phase were sub-cultured in 2 mL RPMI 1640 medium supplemented with 0.0001% human fibronectin (Sigma-Aldrich, St. Louis, MO, USA) for 1 h at 30 °C at 200 rpm. Subsequently, cells were examined microscopically or washed three times with 2 mL PBS before harvesting. Cells were resuspended in 80 μL PBS, with the addition of 20 μL 5× sample loading buffer (with β-ME), boiled for 10 min, and rested on ice for 10 min. The cells were spun down for 10 min, and 90 µL of the supernatant was transferred to a fresh tube prior to immunoblot analysis with specific FN-specific antibody [78].

### 2.8. Adhesion Assay

The adhesion assay was conducted as previously described [77]. In brief, *C. albicans* cells were grown overnight in YPD at 37 °C with 180 rpm agitation. Cells were collected by centrifugation for 5 min at 10,000× g, washed with PBS, and standardized to 5 × 10^7^ *C. albicans* cells/mL in RPMI-1640 medium supplemented with L-glutamine and buffered with MOPS acid. Next, 100 μL (5 × 10^6^ cells) aliquots of the cell suspension were placed in each well of a nonpyrogenic polystyrene flat-bottom 96-well microtiter plate and incubated for 1 h at 37 °C. The wells were washed three times with 10 mM PBS before being quantified by the XTT (2,3-bis-(2-methoxy-4-nitro-5-sulfophenyl)-2H-tetrazolium-5- carboxanilide) reduction assay [79]. Briefly, the adherent cells were incubated with XTT (0.5 mg/mL + 1 μM menadione in Ringer’ solution) in the dark. The absorbance of reduced XTT was measured in a microtiter plate reader at 490 nm, as described previously [47].

### 2.9. Biofilm Formation Assay

To assess the ability of *C. albicans* cells to form biofilm, cells of the strains were prepared as described in the adhesion assay, except that the cells were standardized to 1 × 10^6^ *C. albicans* cells/mL after washing. Then, 200 μL aliquots (2 × 10^5^ cells) of the *C. albicans* cell suspension was placed in the wells of a 96-well microtiter plate and incubated for 48 h at 37 °C before XTT reduction assay.

### 2.10. Spotting Assay

The spotting assays were carried out as previously described [74]. Concisely, cells of the *C. albicans* strains were grown in YPD to the mid-log phase. The cultured strains were diluted to an optical density of 1.0 at OD_600_ (approximately 2 × 10^7^ cells/mL) and then serially diluted from 10^7^ to 10^2^ cells/mL. The diluted cultures were spotted on agar plates at a volume of 5 μL and left to grow.

### 2.11. Cellular Image Observation and Recording

The images of the cultured cells were recorded with a Nikon 50i microscope at 400× magnification. Colonies were photographed with a MEIJI stereoscopic microscope EMZ5 at 40× magnification. The monographs were digitized and processed using Adobe Photoshop software.

### 2.12. Statistical Analysis

Unless stated otherwise, three independent assays were conducted, and each sample was assayed in triplicate. Statistical analyses were performed using GraphPad Prism software, v.8.0 (GraphPad Software, Inc., La Jolla, CA, USA), by one-way analysis of variance (ANOVA), followed by Tukey’s post hoc analysis. The results are expressed as the mean ± standard deviation (SD). The *p* < 0.05 indicate a statistically significant difference. The asterisks used to indicate statistically significant difference are as follows: * *p* < 0.05; ** *p* < 0.01; *** *p* < 0.005; **** *p* < 0.001.

## 3. Results

### 3.1. The Filamentous Growth Caused by the Repressed CaCDC4 Expression Could Be Partially Suppressed by the Constitutive GHP1 Expression in C. albicans

**Figure 1:**
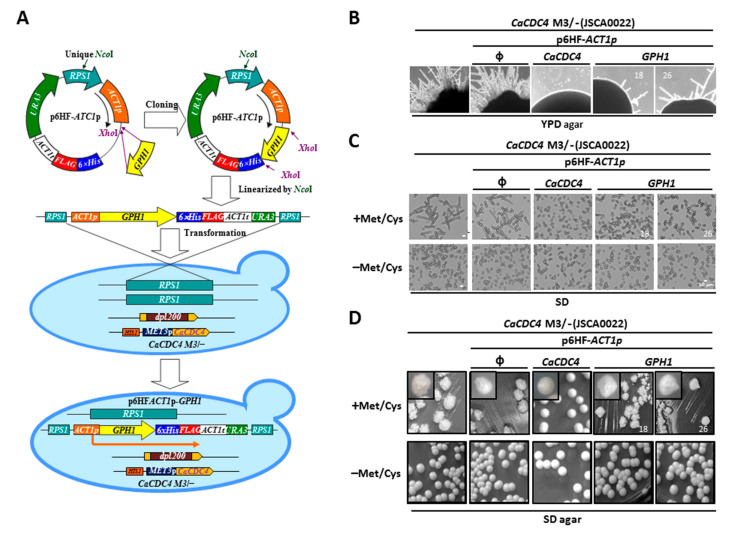
The constitutive expression of *GPH1* suppresses the filamentous mode of growth when the expression of *CaCDC4* is repressed in *C. albicans*. (**A**) The diagram illustrates the strains used. The cells were (**B**) plated on YPD plate or were grown in the SD media (**C**) or plate (**D**) with or without 2.5 mM Met/Cys. The “ϕ” represents empty plasmid p6HF-*ACT1*p. Bars represent 10 μm. “18” and “26” represent different isolates of strains with p6HF-*ACT1*p-*GPH1*.

We previously identified the Gph1 protein as a *C*. *albicans* Cdc4-interactor [48]. To understand the functional link between *CaCDC4* and *GPH1*, a *C. albicans* strain capable of repressing *CaCDC4* expression with methionine and cysteine (Met/Cys) and constitutively expressing *GPH1,* together with those expressing *CaCDC4* and none, were created (Figure 1A). To evaluate the outcome of *GPH1* expression on the filamentous growth of cells with *CaCDC4* expression being repressed, the cells of the above strains, together with their parental strain, were plated onto YPD rich media (Figure 1B) or were grown in the minimum media (Figure 1C) with or without 2.5 mM Met/Cys. After obtaining the constructed strains, we unexpectedly found that the expression of *CaCDC4* of *CaCDC4* M3/− on plates of YPD-rich media appeared to be repressed; hence, they grew as filamentous forms but could mostly be suppressed when constitutively expressing *CaCDC4* from the p6HF-based plasmid (Figure 1B). As expected, the constitutive expression of *CaCDC4* but not the empty plasmid completely suppressed the filamentous mode of growth when the *CaCDC4* expression was repressed (Figure 1C). It appeared that the filaments as a result of the repression of *CaCDC4* expression were mixed with the hyphal and pseudohyphal cells, similar to previous observations by us [40] and others [28]. Significantly, the constitutive *GPH1* expression could somewhat suppress the filamentous development when the *CaCDC4* expression was repressed (Figure 1C). Curiously, the suppression of filamentous growth by *GPH1* on SD plates appeared to be indistinct (Figure 1D). Overall, these results suggest that *GPH1* is functionally related to *CaCDC4* regarding the control morphogenesis and that *GPH1* negatively modulates hyphal formation.

### 3.2. C. albicans Gph1 Protein Being Reduced in the Presence of CaCdc4 May Be the Result of Polyubiquitin-Proteasome-Dependent Degradation

**Figure 2:**
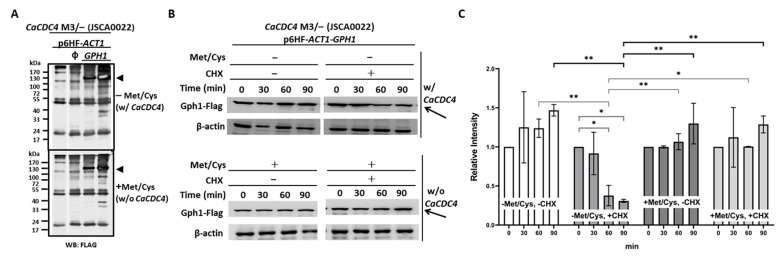
Gph1 protein level decreases in the presence of *Ca*Cdc4 in *C. albicans.* (**A**) Cells of strains, as indicated, were grown in SD with or without 2.5 mM Met/Cys. Triangles indicate the migrated position of Gph1 protein. The “ϕ” represents empty plasmid p6HF-*ACT1*p. (**B**) Cells of strain *CaCDC4* M3/– carrying p6HF-*ACT1-GPH1* were grown in SD with or without 2.5 mM Met/Cys and in the presence or absence of cycloheximide (CHX) for the indicated time. Arrows indicate the Gph1 protein. Cells of all strains were collected and subjected to Western blotting analysis after growing in the indicated condition. SD donates synthetic defined medium. The anti-FLAG antibodies used as the Gph1 are tagged with FLAG. The β-actin used as a loading control was detected by the anti-β-actin antibody. (**C**) To quantify the protein levels, two independent experiments, including the one in (**B**), were used. The Gph1 levels were normalized to those of β-actin and expressed as the relative intensity. Statistical analyses were performed by one way ANOVA, with * *p* < 0.05 and ** *p* < 0.01 as indicated. Representative data from one of the three independent experiments are shown.

Because *GPH1* negatively modulates hyphal development (Figure 1B,C), we presumed that Gph1, like Sol1 [28] and our recently characterized Thr1 [51], is the target of *Ca*Cdc4 and is governed by ubiquitination for degradation. To assess the possible regulation of *Ca*Cdc4 and Gph1, cells of the same strains as in Figure 1 were grown in the minimum media with or without Met/Cys, and the proteins were extracted and subjected to Western blotting analysis. The protein level of Gph1, with or without expression of *CaCDC4*, showed no apparent difference (Figure 2A). However, the de-repressed *CaCDC4* expression led to a reduction of the level of Gph1 protein with translation inhibitor cycloheximide (Figure 2B,C), suggesting that Gph1 protein is targeted for degradation by *Ca*Cdc4. The results indicate that the *Ca*Cdc4 negatively regulates the level of Gph1 protein and that the Gph1 adversely controls filamentation.

### 3.3. Cells Overexpressing or Lacking GPH1 Bear No Morphological Changes but Those without GPH1were Apt to Aggregate for a More Extended Period in Normal Growth Condition

To evaluate the role of *GPH1* in morphogenesis, we made a strain Tet-on-*GPH1* (Supplementary Figure S1A), where the expression of *GPH1* is induced by doxycycline (Dox). The massive induction of *GPH1* expression, both transcriptionally (Supplementary Figure S1B) and translationally (Supplementary Figure S1C), was confirmed. However, no morphological alteration could be observed, suggesting that cells overexpressing *GPH1* were unable to interfere with cellular morphology in *C. albicans* (Supplementary Figure S1D). To further determine whether *GPH1* has a role in morphogenesis, the *CaSAT1*-flipper method (73) was used to create the *GPH1* homozygous null mutant (*gph1*Δ/*gph1*Δ). Southern blotting analyses were used to validate the mutants. As shown in Supplementary Figure S2B, the *Pvu*II-digested genomic DNAs extracted from each of the strains could be detected with a probe specific to the *GPH1* locus flanked with *Pvu*II sites generating the expected sizes (Supplementary Figure S2A). Therefore, we proved that the created mutants were correct. By RT-PCR analyses, as expected, the *GPH1* expression was only observed in the wild-type SC5314 (*GPH1*/*GPH1*), the *GPH1* heterozygous null mutant (*GPH1*/*gph1*Δ), and the *GPH1* complementation strain *gph1*Δ*/gph1*Δ+GPH1, but not in the homozygous null mutant (*gph1*Δ/*gph1*Δ) (Supplementary Figure S2C). The expression level of *GPH1* was approximately two-fold less in *GPH1*/*gph1*Δ) and *gph1*Δ*/gph1*Δ+GPH1 than that of SC5314 (Supplementary Figure S2C). No apparent morphological alteration between the *GPH1* null mutant and the wild-type in the normal growth condition could be found (Supplementary Figure S3A). However, after prolonged incubation, compared with cells of the wild-type, those of the *GPH1* null mutants exhibited an increase in aggregation (Supplementary Figure S3B), suggesting that cells lacking *GPH1* may alter the properties of the cell wall, consequently promoting the cell–cell interaction.

### 3.4. The GPH1 Null Mutant Shows No Growth Defect in Normal Growth Condition and Various Stressful Conditions

To determine if *GPH1* can have a general effect on growth, cells of the *gph1*Δ/*gph1*Δ, together with the *gph1* heterozygous null mutant (*GPH1*/*gph1*Δ), the *GPH1* complement strain *gph1*Δ/*gph1*Δ+*GPH1*, and the wild-type SC5314 (*GPH1*/*GPH1*), were grown in either liquid or semi-solid YPD. Cells lacking *GPH1* showed no growth defect both in YPD liquid medium (Supplementary Figure S4A,B) and YPD plate (Supplementary Figure S4C)*,* suggesting that *GPH1* bears no role in the maintenance of growth. *GPH1* is involved in the synthesis of β-glucan and energy conversion in *S. cerevisiae*; hence, we presumed that this is common in fungi, including *C. albicans*. We set up the spotting assays with conditions including various cell wall damaging agents, different carbon sources, and distinct nutrient-depleted states at either 30 or 37 °C. However, with cells of the wild-type, those of the *GPH1* null mutant showed no consequence in growth ability (Supplementary Figure S5), suggesting that either *GPH1* plays no role or the presence of *GPH1* redundant genes in the cell wall structure and energy conversion. As a result, we have sought alternative assays that may reveal the *GPH1* function.

### 3.5. C. albicans Cells Lacking GPH1 Reduce the Ability to Form Germ Tube in Response to the Hypha-Inducing Condition

**Figure 3:**
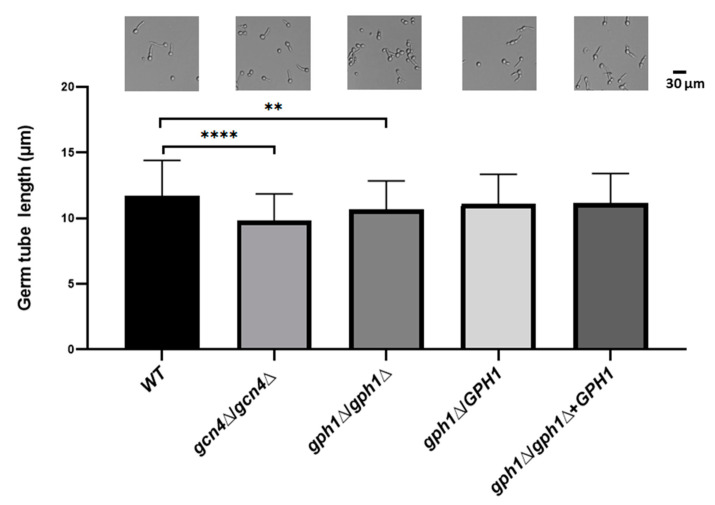
*GPH1* acts positively in germ tube formation in *C. albicans*. The exponentially cultured strains were subjected to 10% fetal calf serum for 1 h to induce germ tube formation. The length of the germ tube was determined from 20 randomly picked germ tube-cells. The representative monographs were from the differential interference contrast (DIC) (also known as Nomarski) microscopy. The *GCN4* null mutant *gcn4*Δ/*gcn4*Δ, known to reduce germ tube formation under the hyphal induction condition, was used as a control. Statistical analyses were performed by one-way ANOVA, with ** *p* < 0.01 and **** *p* < 0.001 as indicated. Representative data from one of the three independent experiments are shown.

While the *GPH1* homozygous null mutant of *C. albicans* did not directly contribute to the yeast-to-hypha transition, we sought to determine the influence of the hyphal induction condition on the mutant. Cells of the *GPH1* homozygous null mutant (*gph1*Δ/*gph1*Δ), together with the *GPH1* heterozygous null mutant (*GPH1*/*gph1*Δ), the *GPH1* complement strain *gph1*Δ/*gph1*Δ+*GPH1*, and the wild-type SC5314 (*GPH1*/*GPH1*), were grown exponentially in YPD and transferred to RPMI 1640 supplemented with 10% fetal calf serum at 37 °C and were subjected to analysis of the length of the germ tube. The ability of the *GPH1* homozygous null mutant to form a germ tube was reduced compared to that of the *GCN4* homozygous null mutant, which is known to be impaired in filamentation under the hyphal induction condition [80] (Figure 3), suggesting that *C. albicans GPH1* is indirectly involved in the yeast-to-hypha transition.

### 3.6. C. albicans Cells Lacking GPH1 Reduce Their Cell Surface Hydrophobicity (CSH)

**Figure 4:**
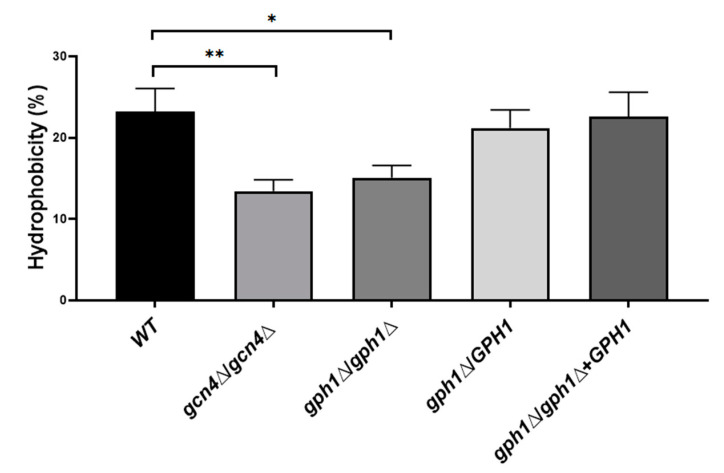
*GPH1* has a role in positively regulating cell surface hydrophobicity in *C. albicans*. The hydrophobicity was measured according to a microbial adhesion to hydrocarbons (MATH) test, as described in the Materials and Methods. All assays are representative of at least three independent experiments performed in triplicate. The original cells read as an absorbance value: A0; the cells left after subjecting to n-hexadecane read as an absorbance value: A1; the hydrophobicity (%) = [1 − (A1/A0)] × 100. The *GCN4* null mutant *gcn4*Δ/*gcn4*Δ, known to reduce biofilm formation, was included in the assay. Statistical analyses were performed by one-way ANOVA, with * *p* < 0.05 and ** *p* < 0.01 as indicated.

*C. albicans* is capable of forming biofilms on abiotic or biotic surfaces. Catheters, dentures, prosthesis (abiotic), and mucosal cell surfaces (biotic) are the most common substrates [81]. Notably, hydrophobic attachment to abiotic and biotic surfaces is also critical in the initial step of biofilm formation, demonstrated by the fact that the adhesion of *C. albicans* to polymeric materials correlates with the cell surface hydrophobicity (CSH) phenotype [82]. Cells of the strains were grown exponentially in YPD and subjected to incubation with hydrophobic molecule n-hexadecane; the percentage of hydrophobicity (CSH) of the cells was determined as the percentage of cells not bound with the n-hexadecane. It appeared that the *GPH1* homozygous null mutant decreased further in CSH compared to that of the *GCN4* homozygous null mutant (Figure 4), which is known to decrease in biofilm formation [83], suggesting that *C. albicans GPH1* is required to maintain CSH in *C. alibicans*.

### 3.7. C. albicans Cells Lacking GPH1 Increase Their Ability to Bind Fibronectin

**Figure 5:**
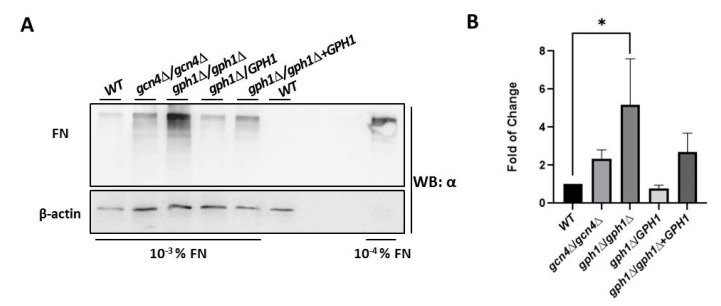
*GPH1* negatively regulates binding with fibronectin (FN) in *C. albicans*. Western blots for FN after pre-treatment of *C. albicans* cells with 10^−3^% fibronectin for 1 h, as described in the Materials and Methods. The *GCN4* null mutant *gcn4*Δ/*gcn4*Δ, known to reduce biofilm formation, was included in the assay. The fibronectin was detected by the antibody specific to fibronectin. The 10^−4^% fibronectin was used as a control. The β-actin was used as a loading control and was detected by an anti-β-actin antibody. (**A**) The represented Western blot. (**B**) The fold of change in association with fibronectin in comparison with the wild-type strain SC5314 (WT). Statistical analyses were performed by one-way ANOVA, with * *p* < 0.05 as indicated.

Adhesion in *C. albicans* refers to the adherence of candida cells to host tissues. It is a phenomenon employing several adhesion proteins (called adhesins, flocculins, or agglutinins) expressed on morphologically changing cell surfaces. Adhesins are agglutinin-like sequences (ALS) that are members of a family of glycosylphosphatidylinositol (GPI)-linked cell surface glycoproteins, capable of binding several extracellular matrix proteins (ECM) of mammalian cells, such as fibronectin (FN), lamin, fibrinogen, and collagen type I and IV [84,85,86,87,88]. To assess the ability of *C. albicans* cells associating with FN, cells of the strains were incubated with FN for 1 h, before washing, harvesting, boiling in the presence of loading dye, and subjecting to Western blotting analysis with a specific anti-FN antibody. It appeared that cells lacking *GPH1* increase the ability to bind FN compared with the wild-type strain, suggesting that *C. albicans GPH1* suppresses the binding with FN (Figure 5).

### 3.8. C. albicans Cells Lacking GPH1 Improve Adhesion Ability but Remain Unchanged in Biofilm Formation

**Figure 6:**
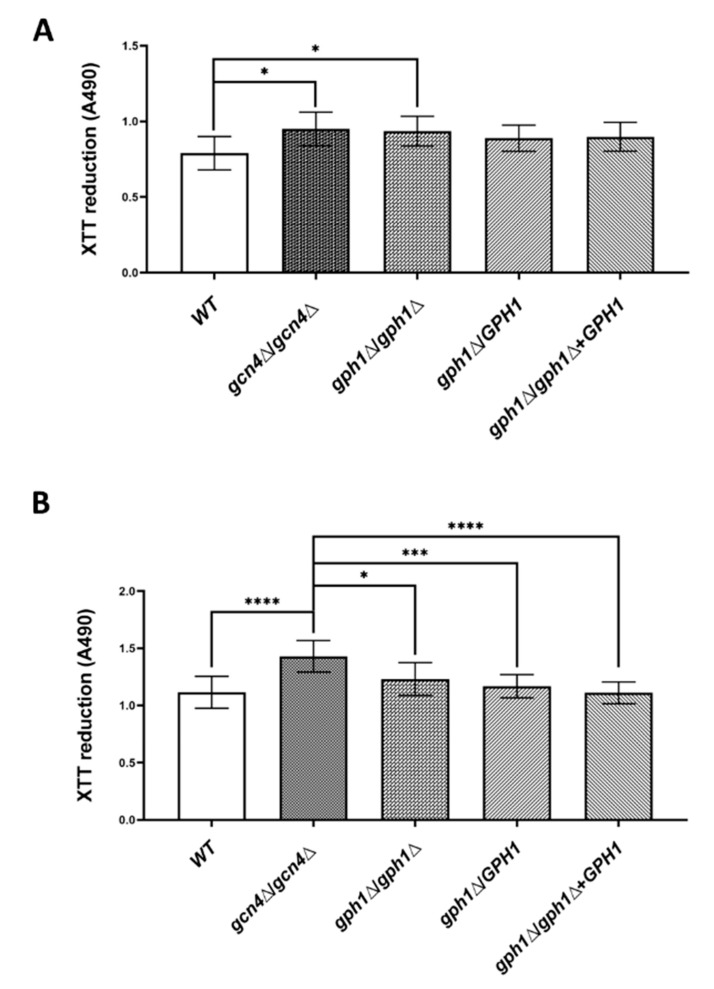
*GPH1* has a role in adhesion but not in biofilm formation in *C. albicans*. Cells of the strains were induced for adhesion in RPMI-1640 medium supplemented with L-glutamine and buffered with MOPS acid as described in the Materials and Methods and were subjected to in vitro XTT reduction assay for adhesion assay (**A**) or biofilm formation assay (**B**). Statistical analyses were performed by one-way ANOVA, with * *p* < 0.05, *** *p* < 0.005, **** *p* < 0.001 as indicated. The *GCN4* null mutant *gcn4*Δ/*gcn4*Δ, known to reduce biofilm formation, was included in the assay.

Because *CaCDC4* negatively regulates biofilm formation, we attempted to ascertain whether *GPH1* plays a similar role. Cells (5 × 10^6^ cells in 100 μL) of the strains were subjected to biofilm induction condition for 1 h at 37 °C in each well of the nonpyrogenic polystyrene flat-bottom 96-well microtiter plate to determine the ability in adhesion assessed by the XTT reduction assay. Cells (5 × 10^6^ cells in 100 μL) of the strains were subjected to biofilm induction condition for 1 h at 37 °C in each well of the nonpyrogenic polystyrene flat-bottom 96-well microtiter plate to assess the ability in adhesion before the XTT reduction assay. As shown in Figure 6A, cells the *GPH1* mull mutants, both the homozygote and heterozygote, and the *GPH1* complement strain increased in adhesion to polystyrene as those of the *GCN4* homozygous null mutant, compared with the wild-type, suggesting that *GPH1* has a role in inhibiting adhesion to the polystyrene. Cells (2 × 10^5^ cells in 200 μL) of the strains were subjected to biofilm induction condition for 48 h at 37 °C in each well of the nonpyrogenic polystyrene flat-bottom 96-well microtiter plate to assess the ability in biofilm formation before the XTT reduction assay. Unlike cells of the *GCN4* homozygous null mutant exhibiting increased ability in biofilm formation, those of all *GPH1*-related strains showed similar ability in biofilm formation (Figure 6B), as compared with those of wild-type, suggesting that *GPH1* does not affect biofilm formation.

## 4. Discussion

In this study, we characterized a *Ca*Cdc4-associated protein Gph1 that had been identified previously [48]. The functional interaction between the Gph1-encoded gene *GPH1* and *CaCDC4* was assessed. Because the F-box and WD40-repeat domains are present in *Ca*Cdc4, we presumed that *CaCDC4* encodes a standard F-box protein of SCF ubiquitin ligase [39] named as SCF*^Ca^*^Cdc4^. We verified that those domains are indispensable for filamentation [40] to control its targets via SCF*^Ca^*^Cdc4^ ubiquitin ligase-dependent degradation. We revealed that the filamentous development due to the repressed *CaCDC4* expression in *C. albicans* was moderately suppressed by the constitutive expression of*GPH1* (Figure 1B,C), which is a positive regulator of filamentous growth. The reason for this can be justified by the hindrance of Gph1 being entirely degraded by the SCF*^Ca^*^Cdc4^ ubiquitin ligase, resembling the degradation of Sol1 blocked in the *Ca*Cdc4-depleted *C. albicans* cells [28]. Indeed, we were able to observe the decreased level of Gph1 when the *CaCDC4* was de-repressed with the translation inhibitor cycloheximide in *C. albicans* (Figure 2B,C). Hence, Gph1 denotes a typical SCF*^Ca^*^Cdc4^ target, which is negatively regulated by *Ca*Cdc4 in a ubiquitin-proteasome-dependent manner.

However, the fact that the *GPH1* expression-induced strain showed no enhancement of filamentous development (Supplementary Figure S1) and the *GPH1* homozygous null mutant could still form filaments under the hypha-induced conditions but with the reduced ability in germ tube formation (Figure 3), suggests that *GPH1* serves no direct role to control yeast-to-hypha transition. The likely reason is that the property of the cell wall of the *GPH1* null mutant has altered, which is evidenced by the improved ability to aggregate in cells lacking *GPH1* (Supplementary Figure S3B); as a consequence, the ability in hyphal formation is affected. The change in cell wall property may alter the structural organization and cell wall layers, which affects the ability in flocculation. We tested if *GPH1* has a role in calcium-dependent self-recognition mediated by adhesins or flocculins [45,89], which has been demonstrated to be required by the *C. albicans CaCDC4* [40]. Cells lacking *GPH1* did not appear to affect the ability in flocculation (Supplementary Figure S6). Hence, the altered cell wall property mediated by *GPH1* has no role in the function of flocculins, including expression and activity, specifically those of Ca^2+^-dependency. The change in cell wall property in *C. albicans* cells lacking *GPH1* was revealed in their decreased cell surface hydrophobicity (CSH) (Figure 4) and increased ability to bind fibronectin (Figure 5), both of which are related to cell adhesion. Of note, while increased CSH [90] and binding ability to fibronectin [91] of *C. albicans* is known to accompany enhanced biofilm formation, *C. albicans* lacking *GPH1* appeared to affect biofilm formation oppositely with regards to CSH and binding to fibronection. Nevertheless, our data indeed demonstrated that the homozygous *gph1* null mutant improves cell adhesion on polystyrene surfaces (Figure 6A). Interestingly, no improvement of biofilm formation was found in the homozygous *gph1* null mutant (Figure 6B). Biofilms form in a sequential process involving adherence of yeast cells to the substrate, proliferation of the yeast cells, development of hyphal or pseudohyphal cells in the upper part of the biofilm, encircled in an accumulated extracellular polymer matrix consisting of proteins and polysaccharides that form a three-dimensional structure with water channels, and finally, dispersion of yeast cells from the biofilm to seed new sites [92,93]. The loss of *GPH1* in *C. albicans* may improve only the initial step of adherence but not the final stage of maturation in biofilm formation.

How the loss of *GPH1* in *C. albicans* cells influences the cell wall property and related functions may be far more complicated than predicted. Of note, the homozygous *gph1* null mutant did not show accumulation of glycogen in *C. albicans* cells (unpublished data), which is inconsistent with the homozygous *gph1* null mutant affecting the no growth defect by the cell wall damaging agents, diverse carbon sources, or the nutrient-depleted conditions (Supplementary Figure S5). We presume that the loss of *GPH1* in *C. albicans* results in the compensation of *GPH1* function related to the character of the cell wall architecture in which the function of many genes in a diverse aspect has interfered. As a consequence, the cell wall is reorganized such that CSH, the ability to bind fibronectin, and adhering to the abiotic surface are altered.

## 5. Conclusions

Our findings indicates that *C. albicans Ca*Cdc4 controls the polyubiquitin- proteasome-dependent degradation of Gph1. While *GPH1* is a positive regulator of filamentation, *GPH1* is negatively controlled by the *CaCDC4*, which suppresses filamentation. *C. albicans* cells lacking *GPH1* affect several features associated with the cell wall structure. Hence, the alterations of these features impact on the adhesion of the early stage of biofilm formation and other related virulent attributes, but not on mature biofilm formation.

## Data Availability

Not applicable.

## Supplementary Materials

The following supporting information can be downloaded at: https://www.mdpi.com/article/10.3390/jof8030233/s1, Figures S1–S6: Candida *albicans GPH1* JoF supplmentary.
