# Supplementary material for: Filament Negative Regulator CDC4 Suppresses Glycogen Phosphorylase Encoded GPH1 That Impacts the Cell Wall-Associated Features in Candida albicans
Source: J Fungi (Basel). 2022 Feb 26;8(3):233. doi: 10.3390/jof8030233 (PMC8949380; doi:10.3390/jof8030233)
Supplement: Supplementary file 1 [file jof-08-00233-s001.zip › jof-1595588-supplementary.pdf]

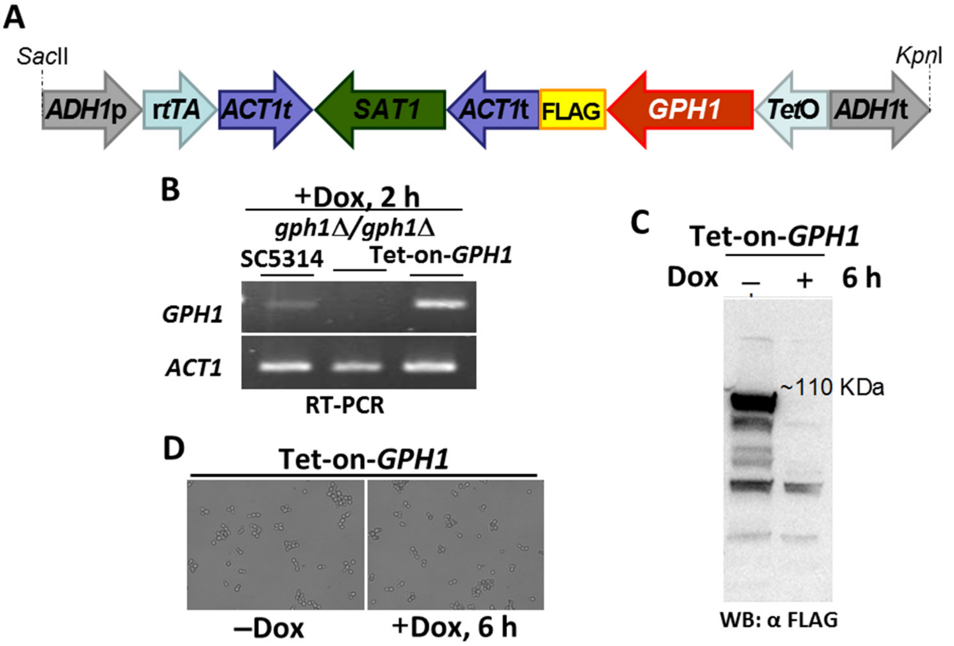

Figure S1. *C. albicans* strain capable of Dox-inducing the expression of *GPH1* has no consequence in cellular morphology. (**A**) Schematic representation of *GPH1* constructed in the Tet-on system. The cassette release from digestion with *Sac*II and *Kpn*I was used to introduce into *C. albicans* cells and to integrate at the *ADH1* locus. (**B**) Dox-induced expression of *GPH1* was assessed by RT-PCR. (**C**) Dox-induced expression of *GPH1* was assessed by the analysis of western blotting. (**D**) Dox-induced expression of *GPH1* microscopically detected no morphological changes in *C. albicans*.

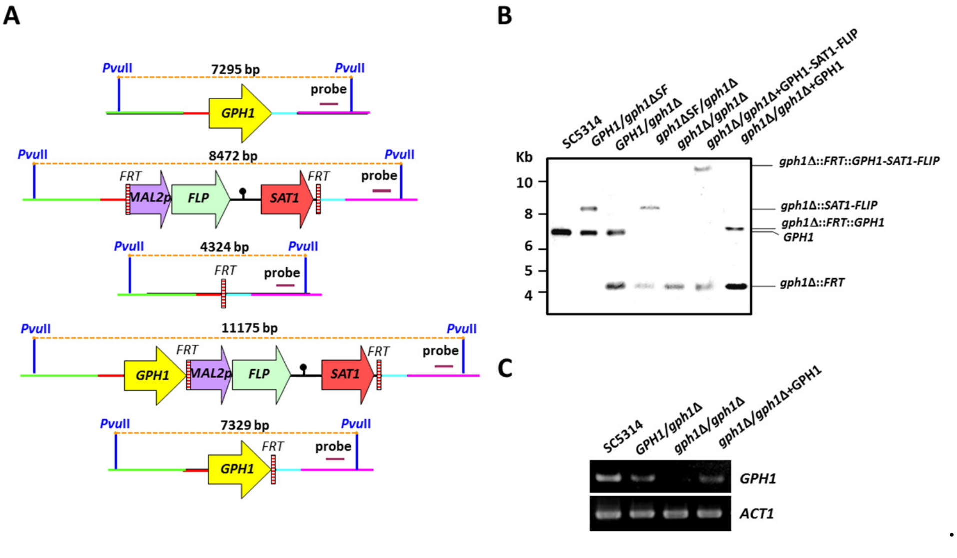

Figure S2. Construction of *C. albicans GPH1* homozygous null mutants using the *CaSAT1*-flipper method. The details of the construction of the strains were described in Materials and Methods. (**A**) The organization of *GPH1* locus with respect to *Pvu*II sites is shown. The relative positions of the probe used and the predicted *Pvu*II- digested pattern of the *GPH1* locus in different strains are indicated. (**B**) Southern blotting analysis. Using a specific probe shown in (**A**), a *Pvu*II fragment of 7295 bp, specific to *GPH1*, could be detected in genomic DNA from SC5314 digested with *Pvu*II; *Pvu*II fragments of 7295 bp and 8472 bp, specific to *GPH1* and *gph1Δ::SAT1-FLIP*, respectively, could be detected in *GPH1/gph1ΔSF*; *Pvu*II fragments of 7295 bp and 4324 bp, specific to *GPH1* and *gph1Δ::FRT*, respectively, could be detected in *GPH1/gph1Δ*; *Pvu*II fragments of 8472 bp and 4324 bp, specific to *gph1Δ::SAT1-FLIP* and *gph1Δ::FRT*, respectively, could be detected in *gph1ΔSF/gph1Δ*; a *Pvu*II fragment of 4324 bp, specific to *gph1Δ::FRT* could be detected in *gph1Δ/gph1Δ*; *Pvu*II fragments of 11175 bp and 4324 bp, specific to *gph1Δ::FRT::GPH1-SAT1-FLIP* and *gph1Δ::FRT*, respectively, could be detected in *gph1Δ/gph1Δ+GPH1-SAT1-FLIP*; and *Pvu*II fragments of 7329 bp and 4324 bp, specific to *gph1Δ::FRT::GPH1* and *gph1Δ::FRT* could be detected in *gph1Δ/gph1Δ+GPH1*. (**C**) RT-PCR evaluation of GPH1 null mutants and the complementation strain. Cells of strains SC5314, *GPH1/gph1Δ*, *gph1Δ/gph1Δ*, and *gph1Δ/gph1Δ*+GPH1 were grown to exponential phase and subjected to RNA extraction, reverse-transcription as described in the Materials and methods.

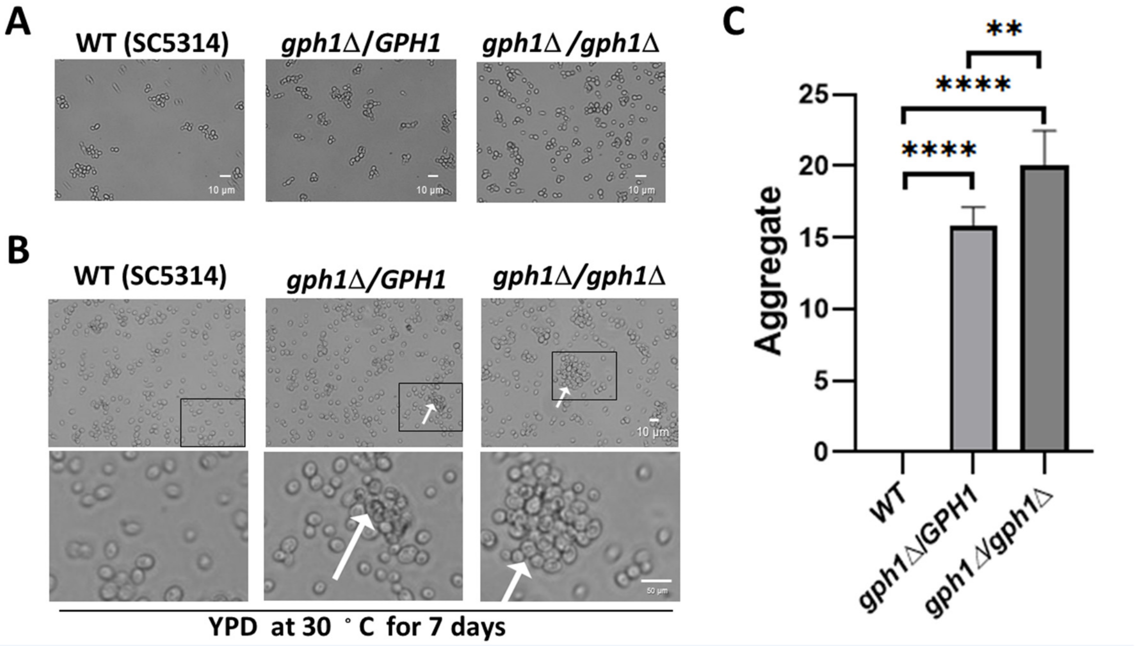

Figure S3. *C. albicans* cells lacking *GPH1* bear no morphological changes but form aggregates after a prolonged time of growth. Cells of the strains in the mid-log phase were grown in YPD (**A**) or were grown in YPD at 30 °C for 7 days and were observed microscopically (**B**). The microscopic images of 100× magnification with aggregates indicated by red triangles (indicative of ageing). The representative aggregates (400× magnification) are indicated by arrows in white, and the enlarged images are from the framed region. Bars represent the size scale, as indicated in µm. (**C**) The quantitative data from two independent experiments are shown. The aggregate is defined by one contains at least 20 cells and determined manually from the enlarged microscopic images. Statistical analyses were performed by one-way ANOVA, with ** $p < 0.01$ and **** $p < 0.001$ as indicated.

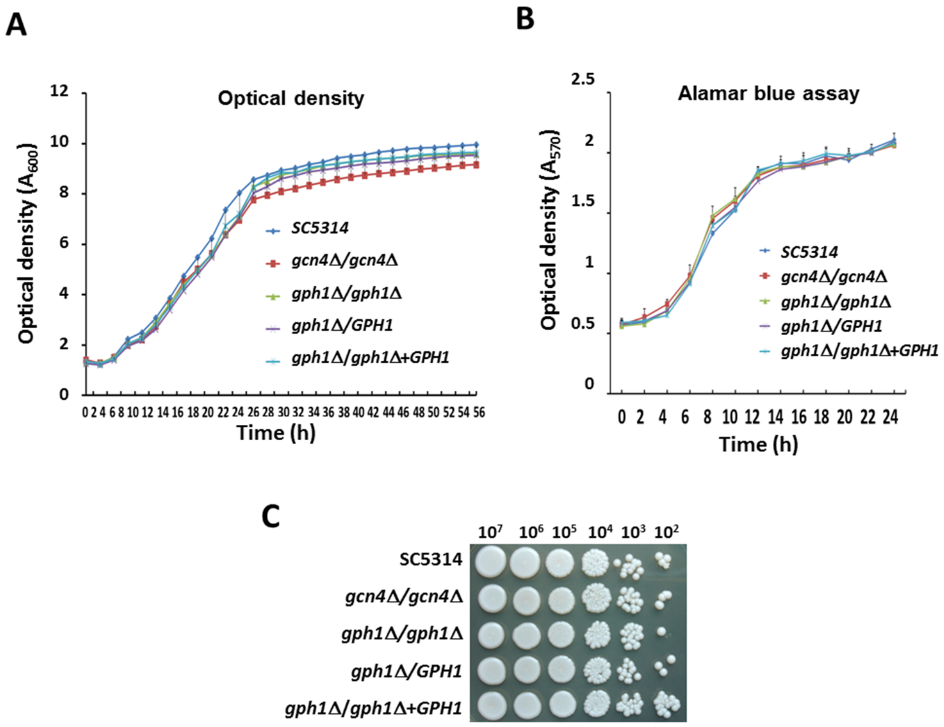

Figure S4. *C. albicans* cells lacking *GPH1* show no defect in growth. The strains were grown in YPD exponentially and diluted into the fresh YPD in an initial $OD_{600} \cong 0.1$. (**A**) The culture was sampled every 2 h up to 56 h and the optical density of the cultured cells was assessed at absorbance 600 nm; (**B**) the cultures were added with Alamar blue sampled every 2 h up to 24 h and the viable cells were assessed at absorbance 570 nm. The curve was determined by two independent experiments, each with triplicates. (**C**) The strains were grown to mid-log phase in YPD, and the cultured cells were subjected to the spotting assay on the YPD plates.

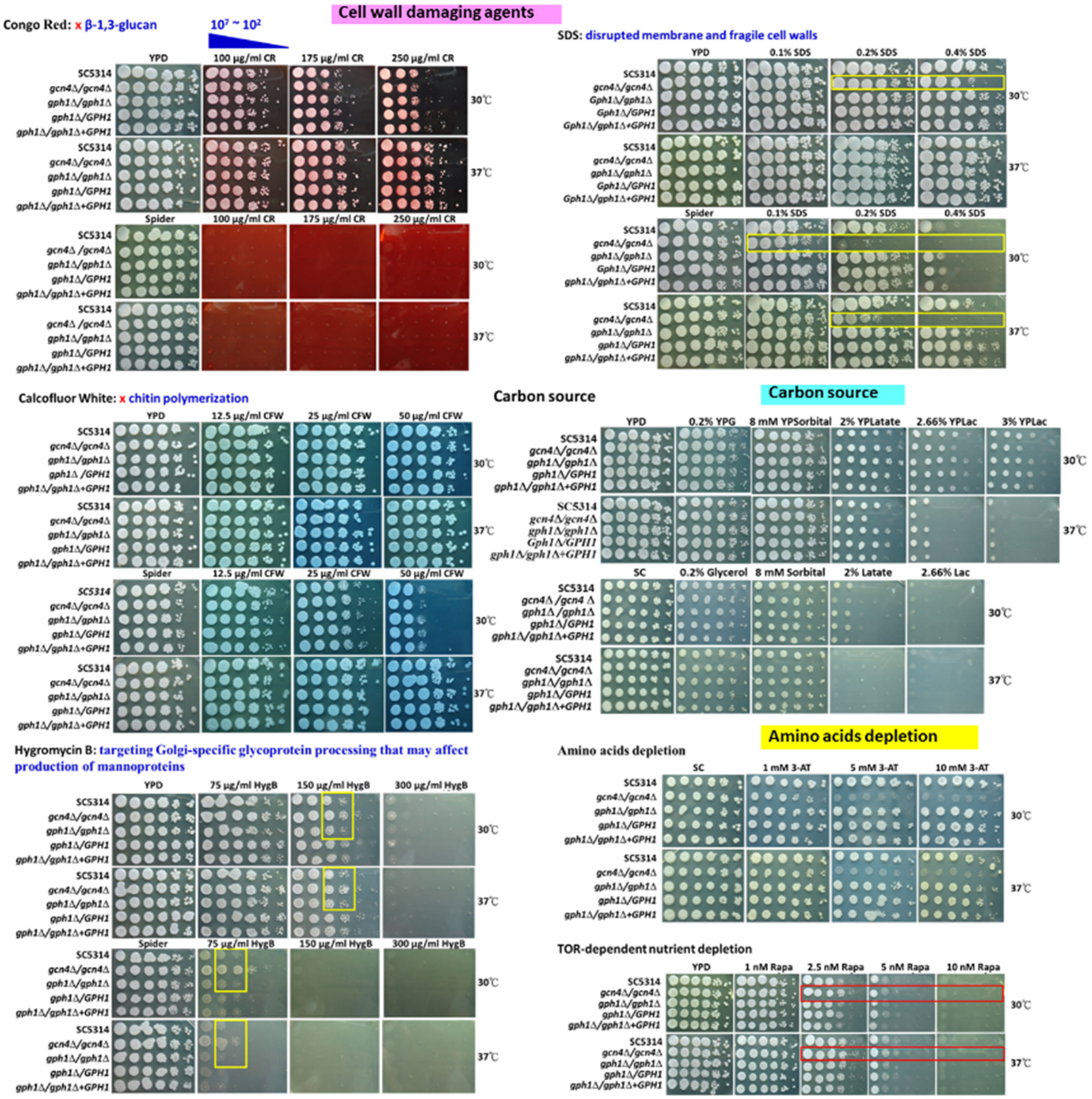

Figure S5. *C. albicans* cells without *GPH1* are not susceptible to cell wall damaging agents, non-glucose carbon sources, and nutrient-depleted conditions. The strains were grown in YPD exponentially and diluted into the fresh YPD in an initial $OD_{600} \cong 0.1$ and were grown further to the mid-log phase. The cultures were subjected to the spotting assay on the plates with indicated agents.

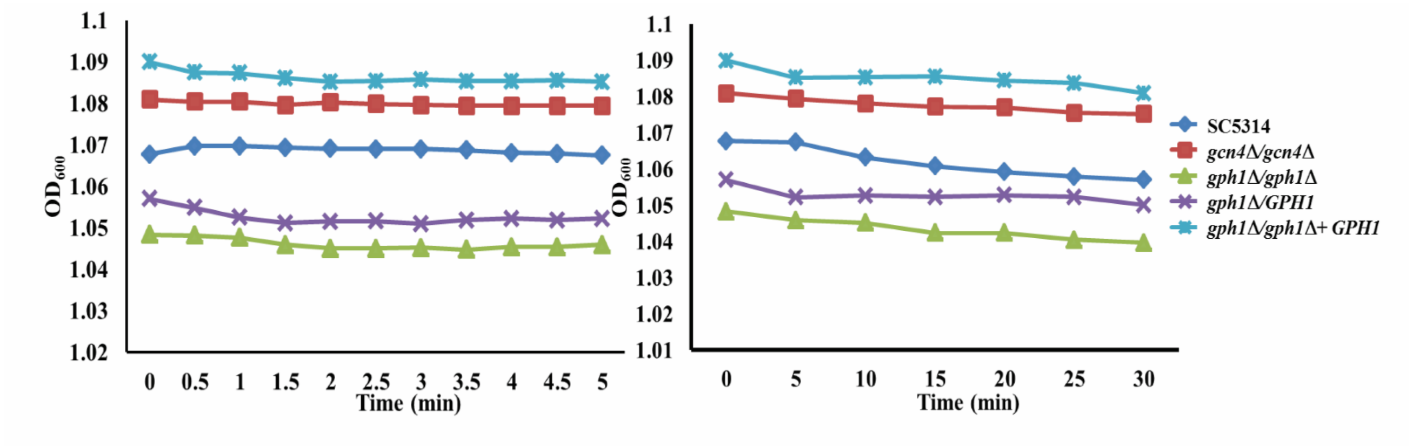

Figure S6. The strains were grown in YPD medium to mid-log phase. The cultures were harvested and washed twice with deflocculation buffer and diluted into the deflocculation buffer to an initial OD600 ≅1.4, followed by initiation flocculation as described in the Materials and Methods. The initial OD600 ≅1.0 after mixing 800 µl deflocculated cell suspension with 200 µl of flocculation buffer (100 mM CaCl2) to initiate flocculation. The absorbance (OD600) is assessed instantly at 30-s intervals for 5 minutes (A) or 5 min for 30 min (B).
